# How Do Spillover Effects Influence the Food Safety Strategies of Companies? New Orientation of Regulations for Food Safety

**DOI:** 10.3390/foods10020451

**Published:** 2021-02-18

**Authors:** Yangchen Xue, Xianhui Geng, Emmanuel Kiprop, Miao Hong

**Affiliations:** 1 College of Economics and Management, Nanjing Agricultural University, Nanjing 210095, China; xyc0903@163.com; 2 School of Business and Economics, Kabarak University, Kabarak 20157, Kenya; ekkiprop5@gmail.com; 3 School of Management and Economics, The Chinese University of Hong Kong (Shenzhen), Shenzhen 518172, China; miaohong@link.cuhk.edu.cn

**Keywords:** food safety, companies strategies, spillover effects, opportunity cost, evolutionary game, food fraud, safety investment

## Abstract

The food safety strategies of companies are a key point in the reduction of food safety risks. In order to encourage the evolution of food safety strategies of companies from food fraud to safety investment, this study builds an evolutionary game model, taking large and small companies as participants, to reveal the dynamic process of spillover effects influencing the choice of food safety strategies of companies. The study shows that (1) the food safety strategies of companies change from safety investment to food fraud, along with the increasing opportunity costs of safety investment. (2) The costs structure of small companies mainly determines whether the industry reaches the equilibrium of safety investment, while the costs structure of large companies mainly determines whether the industry reaches the equilibrium of food fraud. (3) Both competition effects and contagion effects encourage companies to choose safety investment. The more obvious spillover effects of incidents on food safety are, the more likely it is that companies will choose safety investments. (4) Increasing the costs to companies for incidents on food safety and reducing the opportunity cost of safety investment motivates companies to choose safety investment. Consequently, a new orientation of regulations for food safety is formed: the government should allocate different regulatory resources to counteract food fraud behaviors or technologies with a different benefit, should increase the technical costs and costs incurred from committing acts of food fraud, and should expand spillover effects of incidents on food safety.

## 1. Introduction

For the past few years, governments around the world have been working to solve the problem of food safety [1]. Nevertheless, the regulatory system has not provided enough confidence to consumers [2]. As for China, from September 2019 to November 2019, the government investigated and managed 78 thousand cases of food safety violations [3]. Previous problems still exist even as new challenges emerge (Table 1), even under the premise of the continuous improvement of policies, continuous strengthening of regulation and law enforcement, as well as the participation of the public in co-governance. The same phenomenon is happening in the U. S. The outbreak of foodborne diseases has not been significantly reduced by major regulations in the food industry [4]. As a result, seven leading foodborne pathogens account for about 112,000 DALYs annually from foodborne related illnesses acquired in the United States [5]. Even under the highly anticipated certification system, food safety during production has not improved significantly [6].

The company is the first responsible entity for food safety and has the final decision and execution power regarding food safety management. Thus, only through focusing on the food safety management of companies can food safety incidents be reduced. The level of food safety risk is directly influenced by the selection of companies’ safety investment or food fraud strategy. The government is unable to reduce food safety risks using the existing policies and regulations probably because the government did not design them based on consideration of the food safety strategies of companies. For instance, the Chinese government revised “the Food Safety Law” and “the Regulations on the Implementation of the Food Safety Law” in 2018 and 2019, but mainly focused on monitoring and evaluating food safety risks in advance, strengthening inspections in the process, as well as increasing penalties on companies after the incident. All the time, whether in theoretical analysis or policy design, the key point of the content of policies and regulations is always based on two basic variables of food safety: the probability of discovery and the intensity of the punishment [7,8].

In theory and in reality, companies are also partly victims of incidents in regards to food safety. Food production is the foundation for the survival of companies, translating to the fact that, food safety is the lifeline of companies. It is the priority of most companies to reduce food safety risks and choose safety investment. Food safety culture and private standards have been established as a factor in ensuring food safety [9]. This is where the policy is set and shown by senior management to impact all employees. Only a few companies engage in food fraud taking opportunistic actions. However, the “melamine” incident of Chinese dairy products in China found 20 out of 154 companies were guilty [10]. In China, less than 3% of food companies employ HACCP (Hazard Analysis Critical Control Point) systems, it could also be observed that most companies are unmotivated to prevent risks [11]. Whether in China or in any other country, with regards to the same industry, similar incidents on food safety occur frequently, and companies tend to apply the same food safety strategies at the same time. We can conclude that there may be some connection between the food safety strategies of companies.

Recent studies have concluded that incidents on food safety have dire impacts on the entire food industry. This has prompted the analysis of spillover effects (SE) of incidents of food safety [12]. In the classical framework of economic analysis, SE refers to effects that an organization, when carrying out an activity, will not only produce the expected effects of the activity but also have an impact on people or the society outside the organization. It is more likely that SE occurs in the same industry [13,14]. In reality, SE in the food industry could be divided into two forms: competition effects and contagion effects and these two forms often exist simultaneously [15]. In credence goods markets, contagion effects dominate [16]. Therefore, the aftermath of incidences of food safety often influences a company’s performance [17]. The aforementioned connection could be explained by the existence of SE.

Spillover effects provide a new perspective for the study of food safety. This study innovatively considered using SE to explain the selection process of the company’s food safety risk management strategy. And from the government’s point of view, based on SE, this study tried to design and improve a rational system to guide companies to choose safety investment and abandon food fraud. This study also discussed how the food safety strategy of companies is influenced by competition effects and contagion effects. Current studies on SE did not conduct analysis from the perspective of the impact of SE on companies’ decisions but rather it proposes policy recommendations from the industry perspective, encouraging the government or companies to take a multifaceted approach in order to reduce the impact of SE from incidents on food safety [18]. From a consumer perspective, and based on the principles of “Consumer First” or “Safety First”, we were curious if governments should design policies to reduce or increase the impact of spillovers.

This study tried to expand the food safety study to strategic interaction between companies and to promote the evolution of food safety strategies of companies to safety investment from the government’s point of view. According to the theory of SE, combined with the opportunity cost of food safety strategies, this study first analyzed the factors statically that influence the decision of food safety strategies in companies. Later, the study constructed an evolutionary game model, taking large and small companies as participants, to reveal the dynamic process of food safety strategies. Finally, the study discusses the practical application of the conclusions on government regulations for food safety.

## 2. Theoretical Framework

In the food supply system, food generally must go through a series of steps to realize its value [19]. This study categorizes the main participants who are directly involved in food production and consumption and depicts a direct relationship with food safety risks as internal participants (consumers and companies). The participants indirectly involved in food production and consumption and indirectly related to food safety risks are categorized as external participants. As internal participants, companies can reduce food safety risks through various forms of investment in food safety, such as building private standards. As external participants, governments influence the food supply system from the formulation of policies and regulations at the macro-level to the sampling inspection and information release at the micro-level. When market failures and government failures occur, other external participants, for example, media, inspection agencies, certification agencies, industry organizations, and capital markets, also participate in and cooperate with the government to supervise. These two types of external participants complement each other in information, technology, and other aspects; therefore, in both theory and reality, the risk of food safety is reduced to a great extent. Therefore, it can be considered that the level of food safety risk is the result of the behavior of internal and external participants, and it is important to analyze the strategic interactions between the participants.

### 2.1. The Relationship between Food Safety Risks, Food Safety Strategies, and Food Safety Issues

Food is typically exposed to many physical, chemical, and microbiological hazards during its production, processing, transportation, and retailing, and the industry is responsible for delivering safe food to the consumer. However, for the past few years, some artificial risks posed by food fraud gradually became the leading risk to food safety. Aiming at producing food with a certain surface value at lower costs, companies commit food fraud, in a bid to make more profits than if they followed the set regulations. Profit maximization motive often makes companies commit food fraud. On the contrary, in order to reduce food safety risks, some companies are willing to adopt HACCP, private standards, and other ways to increase food safety investment.

**Figure 1:**
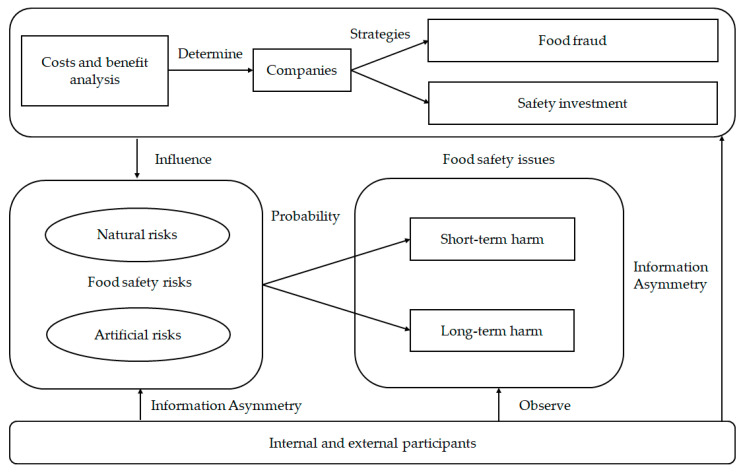
Relationship between food safety risks, companies’ strategies, as well as food safety issues.

In Figure 1, the costs and benefit analysis determined whether companies engage in food fraud or safety investment, and the choice of companies directly influences the level of food safety risk. Owing to the strong asymmetry of food production information, the costs and benefit analysis of companies must take not only the actual costs and benefit of food safety strategies but also the opportunity cost of choosing the safety investment rather than food fraud into consideration. Building private standards is an important method of food safety investment.

In the food supply chain, food safety risks are triggered by a certain probability, which causes food safety issues [20]. Consumers find it difficult to directly observe the food safety issues and production behavior of companies. The benefit drives some companies to commit food fraud in a bid to reduce costs [21]. Researchers have a long concern about this form of moral hazard caused by information asymmetry [22]. Information asymmetry refers to the fact that each person in a transaction has different information. Moral hazard, to put it simply, is the situation that brings greater risk due to opportunistic behavior. However, what needs to be pointed out is that internal and external participants could still attribute food safety issues by observing and judging them, even if there is an asymmetry in the information on risk information and companies’ strategies.

### 2.2. Behavioral Logic of the Food Safety Strategies of Companies

**Figure 2:**
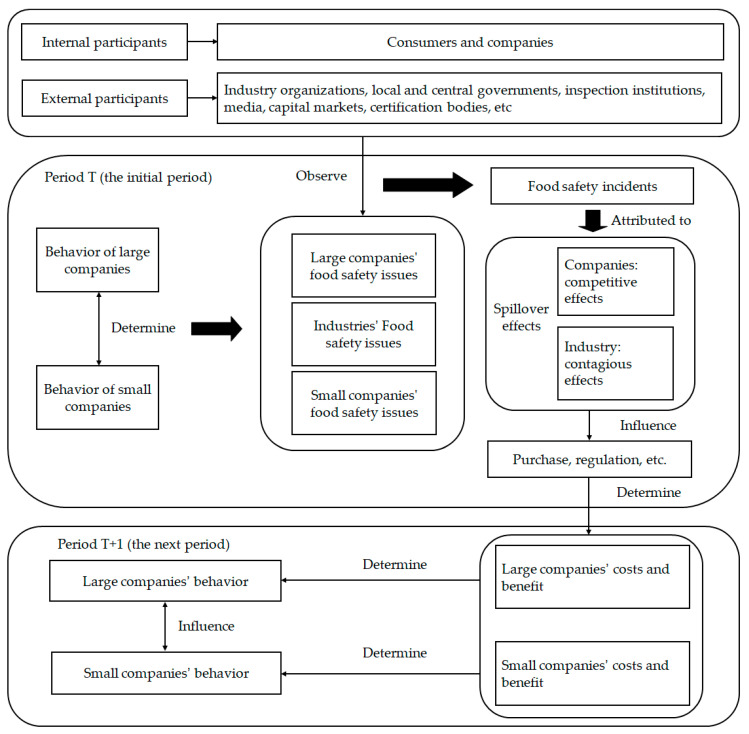
Analysis of the formation mechanism of companies’ food safety strategies.

In the food supply system, there exist several large and small food companies (Figure 2). During period T (the initial period), large and small companies decide on actions of food fraud or safety investment through costs and benefit analysis, which also determines the food safety risk level, and forms the food safety risk level of the whole industry. Correspondingly, large companies, small companies, as well as the entire industry have a certain probability of food safety issues. There are strategic interactions between large and small companies [23,24] due to the indirect and network reciprocal relationships between companies [25].

During period T (the initial period), food safety issues are jointly observed through a variety of methods by internal and external participants. An interpretation is that the incidents on food safety are the objective of the existence of the external performance of food safety risk. In short, the essence of incidents on food safety is the observation of participants of food safety issues, and incidents on food safety produce two main results: (1) these incidents are used as a basis to identify whether the company chose safety investment or food fraud by participants. (2) Due to SE of incidents on food safety, if the participants attribute the incidents to the behavior of the company, there will be competitive effects, in other words, the participants deny the company that perpetrated the incident and affirm other companies in the same industry. On the contrary, if participants attribute the incident to the industry, contagion effects occur, that is to say, the participant denies the entire industry [26]. 

The purchase, investment, regulation, and other behaviors of internal and external participants will change, influenced by incidents on food safety [27]. These changes determine the costs and benefit of food safety strategies for large and small companies in period T + 1 (the next period) and their choice of food fraud or safety investment. Due to SE of the aforementioned incidents on food safety, large and small companies are interdependently taking the actions. Table 2 points out the costs and benefit of food safety strategies of companies.

## 3. Methods and Models

Evolutionary game analysis based on the behavior of participants has become one of the main tools of the analysis of food safety, this is because it can involve government, consumers, companies, and other subjects. Current studies from the perspective of the evolutionary game between the two participants mainly consist of games between the government and companies [28], and between food suppliers and manufacturers [29]; the studies from the perspective of tripartite participants mainly consist of games of governments, companies, as well as consumers [30]. 

Current studies have not thought from the industry perspective on the evolutionary games among heterogeneous companies. Therefore, it is impossible to answer the question of food safety strategies choices of heterogeneous companies when they face different costs and benefit. Evolutionary game theory attaches importance to how cooperation could be generated and spread in disordered groups [31,32] and expanded the traditional game by, for example, relaxing the rational hypothesis, refining the Nash equilibrium, as well as investigating the dynamic adjustment process. The content in this study satisfies the basic assumptions of evolutionary games, for example, the number of game groups at large. Participants in the game could continuously adjust their strategies based on imitation, trial, error, and learning behavior [33]. Thus, we could reasonably construct and analyze the evolutionary game model with large and small companies as participants.

### 3.1. Model Assumptions

Assumption 1. Only two types of game groups exist in the game: large companies (T) and small companies (S) in the same food industry. Individuals in both groups are limited rational people, their strategy choices are based on the actual utility of the strategy, and both sides of the game choose the strategy simultaneously. The game return matrix could be viewed as conforming to the expectation theory, which satisfies the form of U = ∑_i▒(p_i w_i). According to UnicodeMath, ▒ is the concatenation operator. Among them, p_i is the objective probability of an incident *i* on food safety, and w_i is the actual benefits of participants after the incident *i* on food safety.

Assumption 2. An assumption is made in the study that, food safety strategies of large and small companies are divided into two situations: safety investment and food fraud in order to simplify the analysis. That is to say, companies’ food safety strategy is discrete. There are only two strategies: safety investment (H) and food fraud (L). In large companies, the ratio of choosing safety investment strategies is *x*, and the ratio of choosing food fraud strategies is 1−*x*; in small companies, the ratio of choosing safety investment strategies is *y*, and the ratio of choosing food fraud strategies is 1−*y*. The game process is to repeatedly draw a member randomly from two types of infinite groups for a paired game. The learning and strategy imitation of game participants is limited to the group. In these circumstances, it is possible to analyze the replication dynamics and evolutionary stability strategies of the two groups.

Assumption 3. The difference in food safety risk is only related to the food safety strategies of companies. The food produced by the safety investment strategy fulfills the requirements of the controllable risks. By contrast, the food produced by the food fraud strategy falls below or does not fulfill the requirements of controllable risks. The rationality among many participants is complementary, in other words, participants are fully rational in observing incidents on food safety, and use this to identify the company’s behaviors and attributes regarding incidents of food safety. *d* means all the negative impacts of food fraud strategy on all companies in the industry after being identified by the participants. This negative impact from the industry is significantly different for large and small companies because large companies often chase both their own interests and social responsibility. An assumption is that the two types of negative impacts are linearly related, that is to say, if the negative impacts on large companies are D_T, the negative impacts on small companies are D_S = αD_T, where α is the discount coefficients for this influence.

Assumption 4. The strategic choices of the two players in the game have complementary effects. When large and small companies use the strategy (T_H,S_H), the industry has the lowest food safety risks and the lowest probability of food safety issues, participants identify large and small companies’ choice of safety investment. When one player uses food fraud strategies (T_H,S_L) or (T_L,S_H), the food safety risks of the industry increases, and the probability of food safety issues increases; then, participants observe the food safety issues, identify, and attribute them. When large and small companies adopt the strategy (T_L,S_L), the industry has the highest food safety risks and the highest probability of food safety issues, and participants identify that large and small companies committed food fraud. In order to simplify the model, without influencing the conclusion of the game, an assumption is made that, when large and small companies choose safety investment strategies, the benefit obtained from the improvement of their food safety levels are consistent, and the impact on production costs is consistent, and when choosing a food fraud strategy, the impact on production costs is consistent.

Based on the four aforementioned assumptions, this study first builds a food safety strategies benefit matrix (Table 3). For large and small companies, the final result of evolutionary games is to solve the Nash equilibrium with mixed strategies.

The meaning of each parameter is as follows:

*V* represents the gains from the reduction of food safety risks when companies choose safety investment strategies due to the influence of competition effects. It means that, after a company chooses the safety investment strategy, it benefits from the participants’ positive evaluations of the company. Such positive evaluations are from the different strategic choices of the company, resulting in the difference in food safety risks, where V є (0,+∞). It is rare for a single company to obtain participants’ positive evaluation for choosing a safety investment strategy, due to the fact that ensuring food safety is a basic responsibility of the company. 

C_H represents the increased production costs when companies choose the safety investment strategy, that is, the costs that the company pays to reduce risk, where C_H є (0,+∞).

C_L represents the reduced production costs when companies choose food fraud strategy, that is, the costs saved by the company because of food fraud, where C_L є (0,+∞).

ΔC indicates the difference in production costs caused by companies’ choice of the safety investment strategy and food fraud strategy, that is to say, the opportunity cost of companies’ choice of safety investment strategy, where ΔC = C_H + C_L.

*D* indicates that, due to contagion effects, after participants observe incidents on food safety, there is the full negative impact of all companies in the industry on large companies, which is the costs of large companies influenced by industry losses, where D є (0,+∞).

α indicates that due to contagion effects after participants observe incidents on food safety, the proportion of the negative impact of all companies in the industry on small companies, which is the discount coefficient of small companies relative to large companies influenced by the industry loss, where 0 < α ≤ 1.

*L* represents the costs of the company after the participants observe an incident on food safety, and participants identified the food fraud strategy. This loss is mainly divided into two parts: the loss caused through the punishment imposed by the government, and the reduced willingness to pay for the products by consumers. This cost is different for large and small companies: L_1 and L_2 are employed to represent these two costs, where L_1 & L_2 є (0,+∞) and L_1 > L_2 > D > αD.

*r* indicates the probability of incidents on food safety when large companies choose the safety investment strategy and small companies choose the food fraud strategy, where r є (0,1).

*K* indicates the probability of incidents on food safety when large companies choose the food fraud strategy and small companies choose the safety investment strategy, where k ∈ (0,1) and k > r. It is generally believed that the behavior of large companies has a greater impact on the industry. If large companies use strategies to increase risk, incidents on food safety are more likely to occur in the industry.

### 3.2. Model Construction

The following is an evolutionary game model according to replication dynamics.

For large companies, the expected return safety investment strategy is U_TH = − yΔC + (1 − y)(V − ΔC − rD), the expected return of food fraud strategy is U_TL = y(− kL_1 − kD) + (1 − y)(− L_1 − D), and the average return for large companies is U_T = xU_TH + (1 − x) U_TL = x[− yΔC + (1 − y)(V − ΔC − rD)] + (1 − x)[y(− kL_1 − kD) + (1 − y)(− L_1 − D)]

Thus, the replication dynamic equation of large companies is

(1) F(x) = dx/dt= x(U_TH − U_T ) = x(1 − x)(U_TH − U_TL ) = x(1 − x)[V − ΔC + L_1 + (1 − r)D − y(V + (1 − k) L_1 + (1 − r − k)D)]

Equation (1) shows that only if x = 0,1 or y_0 = (V − ΔC + L_1 + (1 − r)D) / (V + (1 − k) L_1 + (1 − r − k)D) , then the proportion of corresponding strategies of large companies is the stationary point of the game.

For small companies, the expected return of safety investment strategy is U_SH = − xΔC + (1 − x)(V − ΔC − αkD), the expected return of food fraud strategy is U_SL = x(− rL_2 − αrD) + (1 − x)(− L_2 − αD), and the average return for small companies is U_S = yU_SH + (1 − y) U_SL = y[− xΔC + (1 − x)(V − ΔC − αkD)] + (1 − y)[x(− rL_2 − αrD) + (1 − x)(− L_2 − αD)]

Thus, the replication dynamic equation of small companies is:

(2) F(y) = dy/ds= y(U_SH − U_SL ) = y(1 − y)(U_SH − U_SL ) = y(1 − y)[V − ΔC + L_2 + (1 − k)αD − x(V + (1 − r) L_2 + (1 − r − k)αD) ].

Equation (2) shows that only if y = 0,1 or x_0 = (V − ΔC + L_2 + (1 − k)αD) /(V − ΔC + L_2 + (1 − k)αD), then the proportion of the corresponding strategies of small companies is the stationary point of the game.

The evolutionary dynamic system composed of Equations (1) and (2) has five stationary points: E_1(0,0), E_2(1,0), E_3(0,1), and E_5 (x_0,y_0 ) . Friedman [34] proposed that for a group dynamic described by a differential equation system, the stability of its equilibrium point could be obtained from the local stability analysis of the Jacobian matrix (*J*) of the system. First, basing on the system composed of Equations (1) and (2), find its Jacobian matrix (*J*), and calculate its determinant (*det J*) and the trace (*tr J*) of the matrix at the aforementioned five stationary points, respectively. Then, determine the sign of *det J* and *tr J* and determine the local stability of the point by the combination of the signs.

For:

(3) J = [■(∂F(x)/∂x & ∂F(x)/∂y @ ∂F(y)/∂x & ∂F(y)/∂y)]= [■(a_11 & a_12 @ a_21 & a_22 ) .

According to UnicodeMath, ■ is the equation-array operator. In this formula:

a_11 = (1 − 2x) [V − ΔC + L_1 + (1 − r)D−y(V + (1 − k) L_1 + (1 − r − k)D)]

a_12 = −x(1 − x)[V + (1 − k) L_1 + (1 − r − k)D]

a_21 = −y(1 − y)[V + (1 − r) L_2 + (1 − r − k)αD]

a_22 = (1 − 2y)[V − ΔC + L_2 + (1 − k)αD − x(V + (1 − r) L_2 + (1 − r − k)αD)]

If the following conditions are met:

① a_11 + a_22 < 0 (Trace condition)

② |■(a_11 & a_12 @ a_21 & a_22 )| = a_11 a_22 − a_12 a_21 > 0 (Determinant condition)

Then the equilibrium point of the replication dynamic equation is asymptotically stable, and this equilibrium point is the evolutionary stability strategy (ESS).

Make b_1 = V − ΔC + L_1 + (1 − r)D, b_2 = V − ΔC + L_2 + (1 − k)αD, b_3 = V + (1 − k) L_1 + (1 − r − k)D, b_4 = V + (1 − r) L_2 + (1 − r − k)αD. Then, Formula (3) could be simplified as:

(4) J = [■((1 − 2x)(b_1 − yb_3) & −x(1 − x) b_3 @ −y(1 − y) b_4 & (1 − 2y)(b_2 − xb_4))] .

When judging the nature of symbols, an inference is mainly made based on some assumptions set by the model and economic principles. Obviously, k < 0.5 < 1− r, r < 0.5 < 1 − k. Based on the sign of b_1, b_2, b_1 − b_3, b_2 − b_4, 16 conditions of dynamic system replication could be obtained. In Table 4, Case 1 has 5 equilibrium points, E_1(0,0) and E_4(1,1) are ESS; Condition 2 has 4 equilibrium points, E_4(1,1) is ESS; and Condition 3 has 4 equilibrium points, E_1(0,0) is ESS. Conditions 4 to 16 are not specifically listed, because, in these conditions, some conditions employ (0,1) or (1,0) as the ESS, and the result of evolutionary stabilization strategies is the strategic differentiation between large and small companies. In some conditions, because V + L_1 + (1 − r)D > k(L_1 − D), V + L_2 + (1 − k)αD > r(L_2 − αD), V + L_1+ (1 − r)D > V + L_2 + (1 − k)αD, k(L_1 + D) > r(L_2 + αD), inequality could not hold, and evolutionary stabilization strategies for large and small companies do not exist. In these 16 conditions, this study focuses more on evolutionary stabilization strategies (T_H,S_H) and (T_L,S_L), for the reason that (T_H,S_H) is the most ideal corporate behavior in reality and (T_L,S_L) is the corporate behavior which needs to be avoided most.

## 4. Results and Discussion

### 4.1. Opportunity Cost of the Companies’ Strategies and Food Safety Strategies Equilibrium

**Proposition** **1.** *As long as the opportunity cost* ΔC *of safety investment is less than the product of the probability of incidents on food safety* r *and the loss of small companies choosing food fraud, both large companies and small companies will choose safety investment.*

**Proof** **of** **Proposition** **1.** In Condition 2, when b_1 > 0, b_2 > 0, b_1 − b_3 = k(L_1 + D) − ΔC > 0, b_2 − b_4 = r(L_2 + αD) − ΔC > 0. There exists an ESS, in which large companies and small companies choose safety investment strategies, and companies that choose food fraud strategies will disappear because of evolution. Condition 2 is equivalent to {■(ΔC < V + L_1 + (1 − r)D @ ΔC < V + L_2+ (1 − k)αD @ ΔC < k(L_1 + D) @ ΔC < r(L_2+ αD))┤ because ${V\;+\;L\_2\;+\;(1\;-\;k)\alpha D\;>\;r(L}_{2}\;+\;\alpha D)$, V + L_1 + (1 − r)D > k(L_1 + D) > r(L_2 + αD), only if $\Delta {C\;<\;r(L}_{2\;}+\;\alpha D)$, the inequality will hold, and both large and small companies with evolutionary stability strategies will choose safety investment strategies. L_2 and αD are part of the costs of small companies, where Result 1 could be drawn. □

**Lemma** **1.** *Follows Proposition 1. For a certain safety investment behavior or certain safety investment technology, whether large companies and small companies choose this behavior or technology simultaneously depends on the cost structure of small companies.*

**Proposition** **2.** *When the opportunity cost ΔC of safety investment lies between the following two items, both large and small companies choose safety investment or both choose food fraud. The first item is the product of the probability of incidents about food safety k and the loss of the large companies choosing food fraud. The second item is the sum of the following three items: the benefit V that the companies obtain from the reduction of food safety risks, the costs paid by the small companies L_2, and the product of the probability that the incident about food safety does not occur 1 − k and the impact of industry losses on small companies*αD.

**Proof** **of** **Proposition** **2.** In Condition 1, when b_1 < 0, b_2 < 0, and b_1 − b_3 = k(L_1 + D) − ΔC > 0, b_2 − b_4 = r(L_2 + αD) − ΔC > 0, there are two evolutionary stable strategies: both large and small companies choose food fraud strategies or both choose safety investment strategies. No matter which strategy the evolution results belong to, companies that choose another strategy will disappear in the process of evolution. Condition 1 is equivalent to {■(ΔC < V + L_1 + (1 − r)D @ ΔC < V + L_2 + (1 − k)αD @ ΔC > k(L_1 + D) @ ΔC > r(L_2 + αD))┤ because V + L_1 + (1 − r)D > V + L_2 + (1 − k)αD, k( L_1 + D) > r(L_2 + αD), only if k(L_1 + D) < ΔC < V + L_2 + (1 − k)αD, the inequality will hold, and both large and small companies with evolutionary stability strategies will choose safety investment or food fraud strategies. □

**Lemma** **2.** *Follows Proposition 2. Whether large and small companies choose safety investment or food fraud behaviors or technologies simultaneously depends on their cost structure.*

**Proposition** **3.** *As long as the opportunity cost of safety investment ΔC is greater than the sum of the following three, large and small companies choose food fraud. The first is the benefit V that the companies obtain from the reduction of food safety risks. The second is the costs L_1 paid by the large companies. The third is the product of the probability that incidents on food safety do not occur 1 − r and the impact of the industry losses on large companies*D.

**Proof** **of** **Proposition** **3.** In Condition 3, when b_1 < 0, b_2 < 0, ${b\_1\;-\;b\_3\;=\;k(L}_{1}\;+\;D)\;-\;\Delta C\;<\;0$, and b_2 − b_4 = r(L_2 + αD) − ΔC < 0, there is an ESS, which both large and small companies choose food fraud strategies, and companies that choose safety investment strategies will disappear in the evolution. Condition 3 is equivalent to {■(ΔC > V+ L_1 + (1 − r)D @ ΔC > V + L_2 + (1 − k)αD @ ΔC > k(L_1 + D) @ ΔC > r(L_2 + αD) )┤ because V + L_1 + (1 − r)D > V + L_2 + (1 − k)αD, V + L_1 + (1 − r)D > k(L_1 + D) > r(L_2 + αD), only if ΔC > V +L_1 + (1 − r)D, the inequality will hold, and both large and small companies with evolutionary stability strategies choose food fraud strategies. L_1 and D are part of the costs of large companies, where Conclusion 3 could be drawn. □

**Lemma** **3.** *Follows Proposition 3**.** For a certain food fraud behavior or certain food fraud technology, whether large and small companies use this behavior or technology simultaneously depends on the cost structure of large companies.*

It is understandable that large companies take the lead in food fraud, and large companies in company with small companies commit food fraud, forming the “Regulatory Captives” or “Industry Crisis”.

**Proposition** **4.** *Follows Lemma 1–3. For a certain safety investment behavior or certain safety investment technology, the food safety strategies of large and small companies change from safety investment to food fraud, as the opportunity cost of the behavior or the technology increases. It could be said that whether companies choose safety investment or not is mainly influenced by the cost structure of small companies and whether they choose food fraud is mainly influenced by the cost structure of large companies.*

As far as the government is concerned, companies may adopt two strategies, namely food fraud and safety investment. Then the government’s responsibility is to adopt new regulatory measures to make all companies adopt safety investment strategies as much as possible, that is, to achieve the ideal equilibrium conditions. In other words, the government could achieve the desired balance by adopting new regulatory measures and changing some of the parameters involved in the Propositions. This is the starting point for the Corollary.

**Corollary** **1.** *Follows Proposition 4. The costs of food safety strategies should be the focus of policy analysis. Different regulatory resources should be allocated to food safety activities or technologies with different costs, and the government should focus more on the regulation of food fraud that causes higher returns to companies. As for food fraud that causes lower benefit, the government could rely on market mechanisms to achieve regulatory goals. Surely, it could eventually achieve the equilibrium outcome of companies choosing to prevent risks or not choosing food fraud through evolutionary games among companies.*

### 4.2. Conditions for Companies to Realize Ideal Food Safety Strategies Equilibrium

In Condition 1, there are two evolutionary stability strategies (T_H,S_H) and (T_L,S_L), where (T_H,S_H) is the most ideal behavior in reality, while (T_L,S_L) is the behavior that needs to be avoided the most. It is necessary to further discuss conditions that could be employed to achieve the strategy of companies’ equilibrium in (T_H,S_H). This is because ΔC > k(L_1 + D) > r(L_2 + αD), the benefit of the ESS (T_L,S_L) is significantly greater than (T_H,S_H), that is to say, the food fraud strategy has become the “Pareto Optimal” of large and small companies. Whereas, the expected direction of the design of policies and regulations is that both large and small companies choose safety investment strategies. Apparently, the optimal companies and the optimal policy are completely opposite, which also explains the strong motivation of food fraud. This target deviation causes the target direction to be exactly opposite to the model direction in the process of analyzing how to reach the ideal equilibrium condition. In order to make policy analysis meaningful, we set the (T_H,S_H) strategy to E_1 (0,0), and the (T_L,S_L) strategy to E_4 (1,1) in the following analysis.

**Figure 3:**
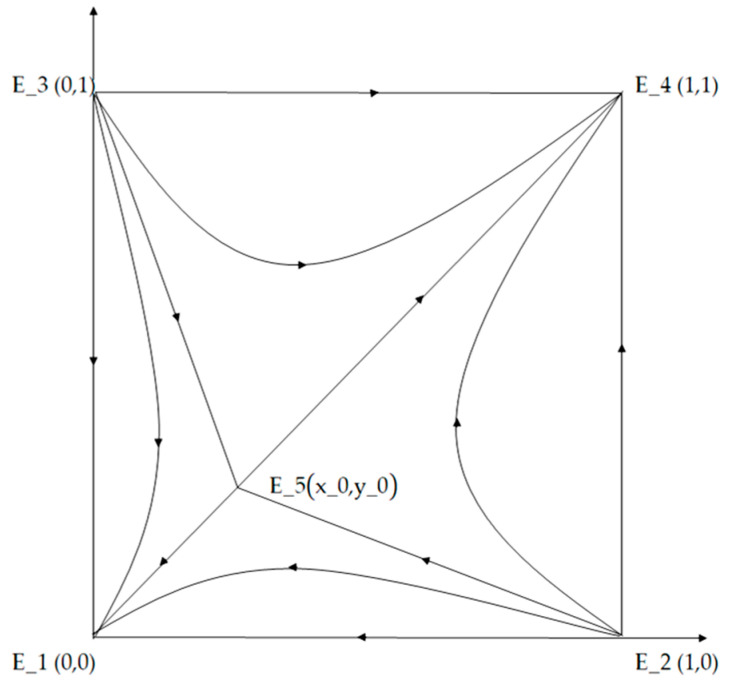
Phase diagram of the dynamic evolution of a five-equilibrium system. Note: E_1 means both large and small companies take strategies of safety investment. E_2 means large companies take strategies of food fraud and small companies take strategies of safety investment. E_3 means large companies take strategies of safety investment and small companies take strategies of food fraud. E_4 means both large and small companies take strategies of food fraud. E_5 (x_0,y_0 ) is the initial state of large and small companies.

As shown in Figure 3, it depicts the dynamic evolutionary game process of large companies group T and small companies group S. In the figure, the polyline E_2 E_5 E_3 formed by two unstable points E_2 & E_3 and saddle points E_5 is the critical line where the system converges to different strategies. The initial state of the system is the proportion of companies that choose different strategies in large company group T and small company group S, respectively. The relative position of the initial state of the system and the saddle points influences the evolution process and stable state of large and small company groups. When the initial state is located in the area to the left of the polyline E_1 E_2 E_5 E_3, the system will converge to E_1 (0,0), and the stability strategy will gradually evolve toward the “Pareto Optimality” direction. Finally, both large and small companies choose safety investment strategies, which is an ideal state. When the initial state is located in the area on the right side of the polyline E_2 E_5 E_3 E_4, the system converges E_4 (1,1). In the end, both large and small companies choose food fraud strategies. Although it has reached an equilibrium state, food safety risks increase and food safety issues increase, which is an undesirable state. Both of the aforementioned states are evolutionary stability closed states, and in either state, participants who take another behavior will disappear in the evolution.

The evolution of the system could not be achieved in one step. Thus, the system will be in a situation where the two strategies coexist for a period of time, due to the choice of different strategies and different proportions by large and small companies. The long-term equilibrium result of the game between the large companies group T and the small companies group S may be the choice of safety investment strategy or food fraud strategy. The specific evolution path and stable state are determined by the area S_(E_1 E_2 E_5 E_3 ) of region E_1 E_2 E_5 E_3 and the area S_(E_2 E_5 E_3 E_4 ) of region E_2 E_5 E_3 E_4. If S_(E_2 E_5 E_3 E_4 ) > S_(E_1 E_2 E_5 E_3 ), the system will be more likely to evolve along the path E_5 E_4 toward food fraud; if S_(E_2 E_5 E_3 E_4 ) < S_(E_1 E_2 E_5 E_3 ), the system will be more likely to evolve along the path E_5 E_1 toward safety investment; if S_(E_2 E_5 E_3 E_4 ) = S_(E_1 E_2 E_5 E_3 ), the probability of choosing safety investment is equal to the probability of choosing food fraud and the direction of system evolution can not be determined. Based on Figure 3, the area of the region E_1 E_2 E_5 E_3 is S_(E_1 E_2 E_5 E_3 ) = 1/2(x_0 + y_0 ). We employ a comparative static analysis method to analyze the influence of each parameter on the area. Suppose a parameter variable to be investigated is θ, then the change rate of the area of this parameter is

(5) (∂S_(E_1 E_2 E_5 E_3 )) / ∂θ = (∂S_(E_1 E_2 E_5 E_3 )) / (∂x_0 ) ×(∂x_0) / ∂θ + (∂S_(E_1 E_2 E_5 E_3 )) / (∂y_0 ) × (∂y_0) / ∂θ =1/2((∂x_0) / ∂θ + (∂y_0) / ∂θ) .

**Proposition** **5.** *When a company chooses safety investment, due to competition effects, the higher the profit from the improvement of its food safety level, the more incentive the companies have to choose safety investment.*

**Proof** **of** **Proposition** **5.** Basing on the *X* and *Y* coordinates of point E_5, ∂x_0 / ∂V > 0 and ∂y_0 / ∂V > 0. Based on the phase diagram, the area formula of S_(E_1 E_2 E_5 E_3 ), and the rate of change formula, ∂S_(E_1 E_2 E_5 E_3 ) / ∂V > 0, and the proposition is proven. □

**Proposition** **6.** *After incidents on food safety, due to contagion effects, the greater the impact of industry losses on a company, the more incentive the companies have to choose safety investment.*

**Proof** **of** **Proposition** **6.** Based on the *X* and *Y* coordinates of point E_5, ∂x_0 / ∂D > 0, ∂y_0 / ∂D > 0, ∂x_0 / ∂α > 0, and ∂y_0 / ∂α = 0. Based on the phase diagram, the area formula of S_(E_1 E_2 E_5 E_3 ), and the rate of change formula, ∂S_(E_1 E_2 E_5 E_3 ) / ∂D > 0 and ∂S_(E_1 E_2 E_5 E_3 ) / ∂α > 0, and the proposition is proven. □

**Lemma** **4.** *Follows Propositions 5 and 6. Whether competition effects or contagion effects, they will increase the probability that the industry produces food toward the direction of safety investment, in other words, the more obvious SE of incidents on food safety, the more likely for small and large companies in the industry to choose safety investment.*

**Corollary** **2.** *Follows Lemma 4. Various measures should be taken to increase SE of incidents on food safety, so as to achieve increasing the probability that the industry will choose safety investment.*

**Proposition** **7.** *After incidents on food safety, the higher the costs the companies pay after the food fraud is identified by the participants, the more incentive the companies have to choose safety investment.*

**Proof** **of** **Proposition** **7.** Based on the *X* and *Y* coordinates of point E_5, ∂x_0 / ∂L_1 = 0, ∂y_0 / ∂L_1 > 0, ∂x_0 / ∂L_2 > 0, and ∂y_0 / ∂L_2 = 0. Based on the phase diagram, the area formula of S_(E_1 E_2 E_5 E_3 ), and the rate of change formula, ∂S_(E_1 E_2 E_5 E_3 ) / ∂L_1 > 0 and ∂S_(E_1 E_2 E_5 E_3 ) / ∂L_2 > 0, and the proposition is proven. □

**Corollary** **3.** *Follows Proposition 7. The government should increase the costs of the company after it is identified as committing food fraud, so as to achieve prompting the industry to choose safety investment.*

**Proposition** **8.** *The lower the opportunity cost of safety investment, the greater the incentive for the companies to choose safety investment.*

**Proof** **of** **Proposition** **8.** Basing on the *X* and *Y* coordinates of point E_5, ∂x_0 / ∂ΔC < 0, and ∂y_0 / ∂ΔC < 0. Based on the phase diagram, the area formula of S_(E_1 E_2 E_5 E_3 ), and the rate of change formula, ∂S_(E_1 E_2 E_5 E_3 ) / ∂ΔC < 0, and the proposition is proven. □

**Corollary** **4.** *Follows Proposition 8. The government should increase investment in the study and development of safety investment technology, take measures to reduce the costs of safety investment strategies, or increase the costs of technology necessary in the reduction of food fraud, in order to prompt the industry to choose safety investment.*

## 5. Conclusions

A continuous game and interactions between large and small companies form food safety strategies of companies. It is a complex and dynamic process between the two parties in the game. According to the theory of SE and the concept of opportunity cost, this study constructed an evolutionary game model and draws many important conclusions. Firstly, the opportunity cost of safety investment behaviors or technologies influences the food safety strategies of companies. Secondly, regarding whether competition effects or contagion effects will prompt companies to choose safety investment, the more obvious SE of incidents on food safety is, the more likely that companies will choose safety investment. Other conclusions could also be drawn. Both large and small companies choose safety investment that is mainly influenced by the cost structure of small companies, and both choose food fraud that is mainly influenced by the cost structure of large companies. There are positive incentives, increasing the costs that companies pay after food fraud is identified, as well as reducing the opportunity cost of safety investment, both of which incentivize companies to choose safety investment. To realize the goal of reducing food safety risks and to solve the food safety problem, the government should intervene and regulate the market from a new orientation, as a critical external participant.

Firstly, different regulatory resources should be allocated to different costs of food safety behaviors or technologies, and the government should pay more attention to monitoring behaviors or technologies of food fraud that provide higher benefit to the companies. This conclusion is derived from Lemmas 1–3, Propositions 1–4, and Corollary 1. As for behaviors or technology of food fraud that brings lower benefit, the government could employ market mechanisms to achieve regulatory goals. Consequently, the costs of food safety behavior or technology should be the key to policy study. Taking precautionary measures to manage potential hazards is often effective [35], and people widely believe that economic evaluation of food safety measures is a critical tool for companies and government performance evaluation [36]. However, whether it is the Food and Agriculture Organization of the United Nations (FAO), or the Chinese Food Safety Law, they ignore prior economic evaluations of food safety risk management behaviors or technologies and treat prior food safety risk assessments as a mere question in the field of natural sciences, which believes that food safety risk assessment is mainly about HACCP. The conclusion of this study reveals that governments should also analyze the costs of food safety behavior or technology of companies. Food safety strategies behavior or technology is not only the concern of natural science but also social sciences. Only by comprehensively evaluating the natural and social attributes of hazards and behaviors can the government conduct targeted food safety management.

Secondly, the public investment in safety investment technology study and development should be increased, the costs of safety investment should be reduced, and the use costs and technological costs of food fraud should be increased, in order to promote the industry choosing safety investment. This conclusion is derived from Proposition 8 and Corollary 4. Food safety has “quasi-public goods” attributes; this means that government should be the main body to increase the investment in the study and development of safety investment technologies. Increased public input from the government can reduce the costs for companies to build private standards. Article 11 of China’s Food Safety Law mentions some of them. However, the cognition of the government towards increasing the costs of food fraud is not comprehensive. To be specific, the government could not only increase the technical costs of food fraud but also increase the costs of the usage of food fraud. “Melamine” of dairy products is an example, if the technical costs of synthesizing melamine are much higher than the production costs of milk powder, or it is hard to obtain melamine, there is no incentive for companies to add melamine to milk powder. The government could refer to hazardous material management, and supervise some low-costs food fraud from the source.

Thirdly, starting with the “Safety First” or “Consumer First” principle, the government should not reduce SE of food safety incidents. Instead, the government should increase the SE of incidents on food safety, in order to increase the probability that the industry will choose safety investment. This conclusion is derived from Lemma 4, Propositions 5–6, and Corollary 2. From the perspective of the industry, food safety incidents are mainly manifested as “Fall Together” contagion effects, which have a significant negative impact on the industry. From the perspective of the government and that of consumers, spillover effects of food safety incidents are more similar to a “Collective Punishment Mechanism”. There are joint responsibilities because the companies involved monitor each other through contractual relationships, hoping for the result of a win-win situation rather than a lose-lose scenario. This joint responsibility between companies in the same industry creates the demand for industry associations, which restrict the behavior of companies in the industry through industry norms, to reduce the collective punishment of the society toward the industry [37]. In terms of policy design, the active role of industry associations in food safety governance should be emphasized. In addition, governments could encourage companies to build private standards by which consumers can distinguish between different companies.

Fourthly, the penalties for companies that commit food fraud should be increased, in order to prompt the industry to choose safety investment. This conclusion is derived from Proposition 7 and Corollary 3. This policy has a clear orientation: companies’ incurring a lot of costs following the acts of failing to meet food safety requirements and committing acts of food fraud must be amplified. These costs include but are not limited to “vote with feet” from internal participants (consumers) and penalties from external participants (such as the government). What is noteworthy is that this conclusion is according to the assumption that internal and external participants could easily observe incidents on food safety, that is to say, the degree of information asymmetry of participants regarding incidents of food safety is relatively low. Therefore, this requires more risk information exchange between internal and external participants, especially about the information of incidents on food safety. The government should play its role in risk information exchange, and promptly report incidents of food safety.

The conclusion of this study is mainly aimed at the subdivided food industries with a large number of companies and the inability of the government to fully supervise. As mentioned above, an essential assumption of evolutionary game theory is that there are many participants. 

## Figures and Tables

**Table 1:** Examples of the most concerning food fraud incidents in China from 2005 to 2019.

No.	Incidents	First Report	Recent Report	Severity of Major Consequence
1	Aquatic product “Malachite Green”	2005	2018	Long-term harm
2	Livestock Products “Sudan Red”	2006	2013	Long-term harm
3	Dairy Products “Melamine”	2008	2008	Illness and Death
4	Edible Oil Product “Ditch oil”	2010	2019	Long-term harm
5	Livestock and Poultry Products “Clenbuterol”	2011	2019	Long-term harm
6	Health products, Liquor “Plasticizer”	2011	2019	Long-term harm
7	Dairy Products “Industrial Gelatin”	2011	2019	Long-term harm
8	Livestock Products “Saccharin”	2013	2019	Long-term harm
9	Livestock Products “Zombie Meat”	2015	2019	Long-term harm
10	Takeaway Products “Unhygienic Production”	2016	2019	Illness
11	Takeaway Product “Inferior Cooking Bag”	2018	2019	Long-term harm
12	Bee Products “Fake Honey”	2018	2019	Long-term harm

Note: The authors sought the data from relevant news and government notices. “First Report“ means adverse effects first identified and “Recent Report” means hazards removed from the market. The long-term harm implies some adverse health effect, perhaps chronic but here it may mean economic loss over a period of time.

**Table 2:** Costs and benefit of food safety strategies of companies.

	Food Fraud	Orientation of Influence	Safety Investment	Orientation of Influence
Costs analysis	Industry losses after participants identify companies’ behavior	Rise	Production costs of food	Rise
	Direct companies’ losses (e.g., government penalties) after participants identify companies’ behavior	Rise		
	Indirect companies’ losses (e.g., reduced willingness to pay by consumers) after participants identify companies’ behavior	Rise		
Benefit analysis	Production costs of food	Fall	Benefit from fulfilling social responsibility of food safety	Rise
			Benefit from capital investment as well as product premiums	Rise

**Table 3:** Benefit matrix of food safety strategies for large and small companies.

Large Companies	Small Companies	
	Safety Investment (*y*)	Food Fraud (1−*y*)
Safety investment (*x*)	− ΔC, − ΔC	V − ΔC − rD, − rL_2 − αrD
Food fraud (1−*x*)	− kL_1 − kD, V− ΔC− αkD	− L_1 − D, − L_2 − αD

**Table 4:** Conditions of the system of replication dynamics and equilibrium point.

	Symbol of b_1	Symbol of b_2	Symbol of b_1 − b_3	Symbol of b_2 − b_4	Number of Equilibrium Points	ESS
Condition 1	-	-	+	+	5	(0,0),(1,1)
Condition 2	+	+	+	+	4	(1,1)
Condition 3	-	-	-	-	4	(0,0)

## Data Availability

The datasets generated and/or analysed during the current study are not publicly available due data are not public but are available from the corresponding author on reasonable request.
